# Supplementary material for: A potential role for acyl-phosphate in the coordination of phospholipid and lipopolysaccharide synthesis in Escherichia coli
Source: bioRxiv. 2026 Mar 14:2026.03.13.711678. Preprint. [Version 1] doi: 10.64898/2026.03.13.711678 (PMC13060810; doi:10.64898/2026.03.13.711678)
Supplement: Supplement 1 [file NIHPP2026.03.13.711678v1-supplement-1.pdf]

bioRxiv preprint doi: https://doi.org/10.64898/2026.03.13.711678; this version posted March 14, 2026. The copyright holder for this preprint (which was not certified by peer review) is the author/funder, who has granted bioRxiv a license to display the preprint in perpetuity. It is made available under aCC-BY 4.0 International license.

SUPPLEMENTAL MATERIAL FOR:

A potential role for acyl-phosphate in the coordination of phospholipid and lipopolysaccharide synthesis in *Escherichia coli*

Tanner G. DeHart[1†], Elayne M. Fivenson[1†], Vincent de Bakker[1], Nazgul Sakenova[1], and Thomas G. Bernhardt[1,2]

[1]Department of Microbiology, Harvard Medical School, Boston, MA, USA

[2]Howard Hughes Medical Institute, Harvard Medical School, Boston, MA, USA

[†]These authors contributed equally to this work

*To whom correspondence should be addressed
Thomas G. Bernhardt
Harvard Medical School
Department of Microbiology
Boston, MA 02115
e-mail: thomas_bernhardt@hms.harvard.edu

bioRxiv preprint doi: https://doi.org/10.64898/2026.03.13.711678; this version posted March 14, 2026. The copyright holder for this preprint (which was not certified by peer review) is the author/funder, who has granted bioRxiv a license to display the preprint in perpetuity. It is made available under aCC-BY 4.0 International license.

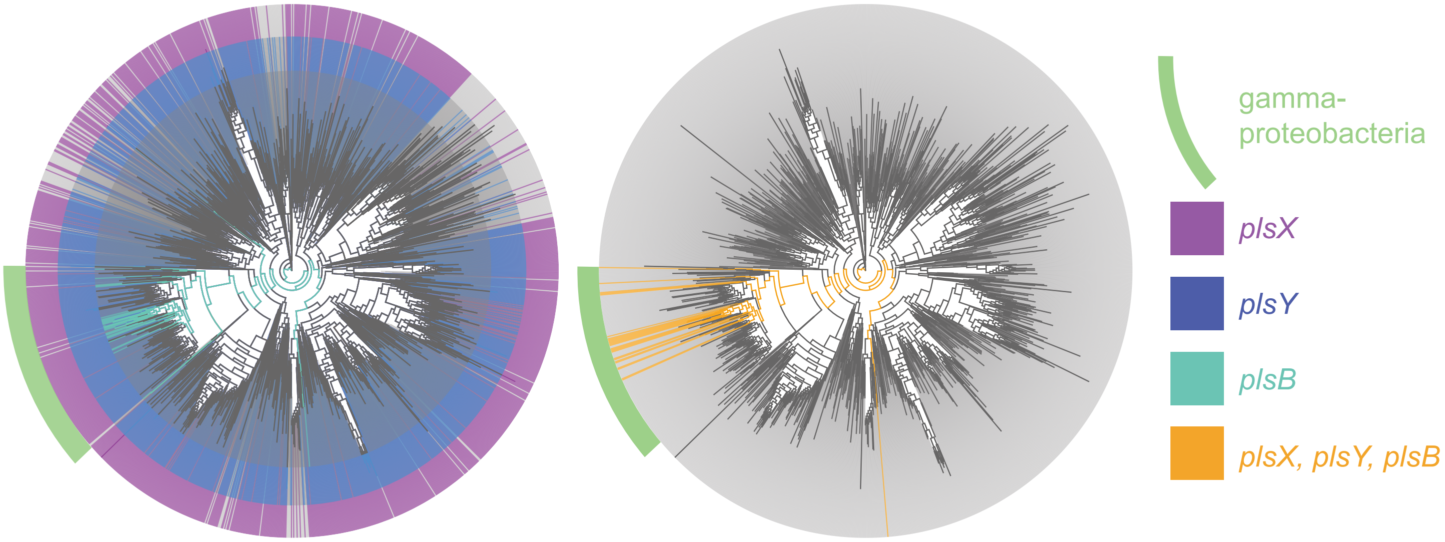

Figure S1: Conservation of phospholipid synthesis genes across bacteria. Each line represents an order of bacteria. Orders within the bacterial class gamma-proteobacteria are shown by the green arc. **Left.** The presence of *plsX*, *plsY*, *plsB* in each genome is represented by purple, blue, or teal, respectively. **Right.** Species that possess *plsX*, *plsY*, and *plsB* are shown highlighted in orange. This figure was made with AnnoTree (1).

bioRxiv preprint doi: https://doi.org/10.64898/2026.03.13.711678; this version posted March 14, 2026. The copyright holder for this preprint (which was not certified by peer review) is the author/funder, who has granted bioRxiv a license to display the preprint in perpetuity. It is made available under aCC-BY 4.0 International license.

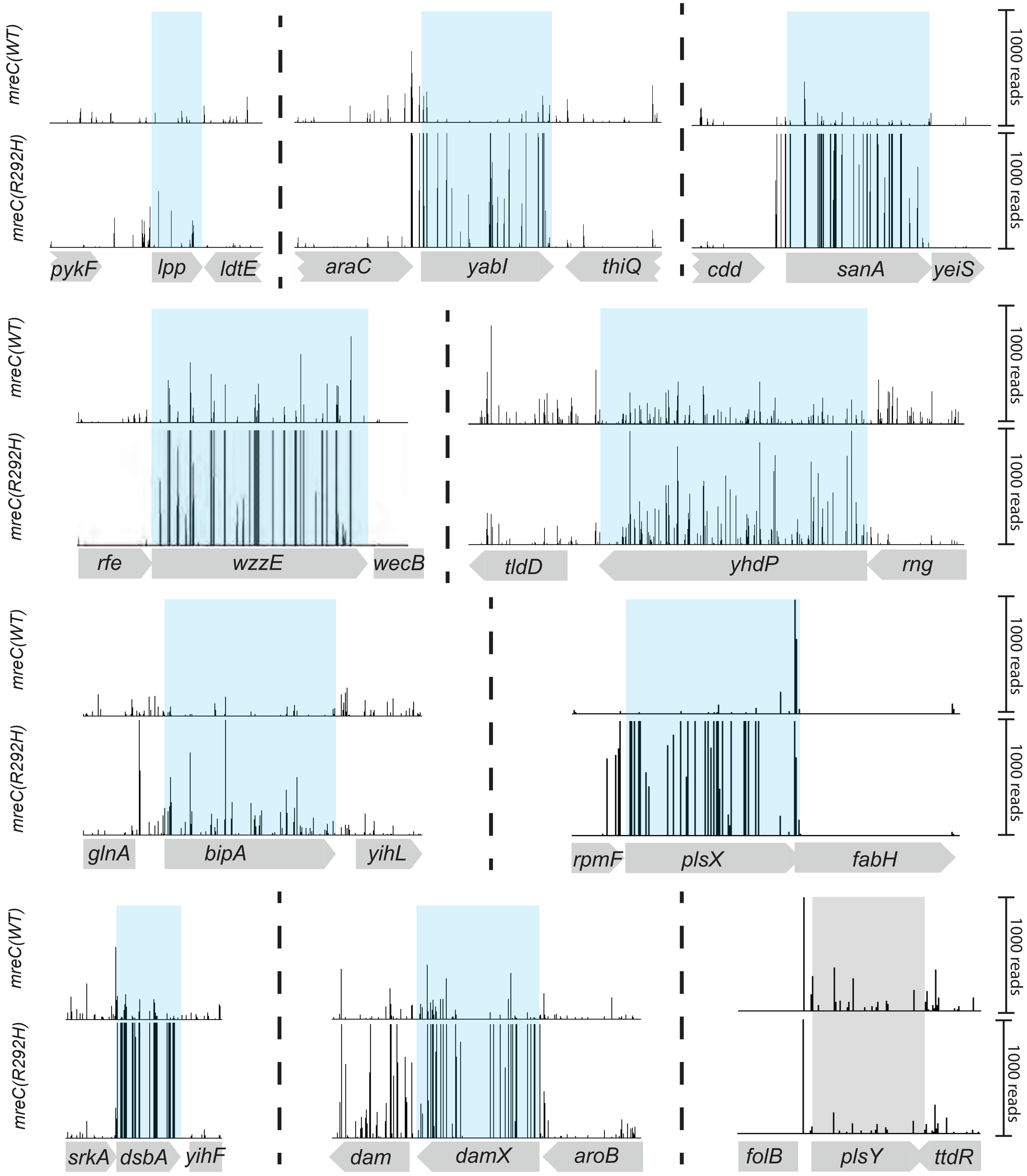

Figure S2: Transposon insertion profiles of genes identified as hits. Tn-Seq insertion profiles for the indicated genes in MG1655 cells expressing *mreC(R292H)* from pPR49 [P_{tac}::*mreC(R292H) mreD*] relative to those expressing *mreC(WT)* from pPR11[P_{tac}::*mreC(WT) mreD*]. The relative height of each vertical line indicates the frequency of insertions at that site in the population. The profile of *plsY* is highlighted in gray to indicate that it was not called at a hit. It is shown for comparison with the *plsX* profile.

bioRxiv preprint doi: https://doi.org/10.64898/2026.03.13.711678; this version posted March 14, 2026. The copyright holder for this preprint (which was not certified by peer review) is the author/funder, who has granted bioRxiv a license to display the preprint in perpetuity. It is made available under aCC-BY 4.0 International license.

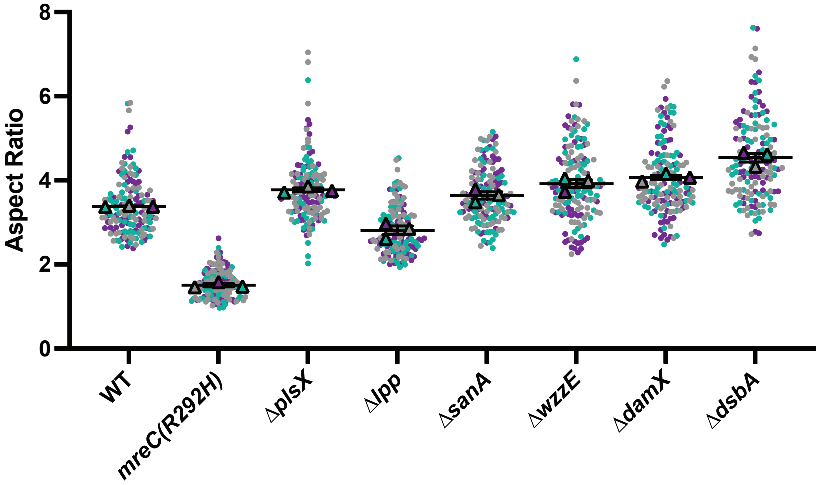

Figure S3: Cell aspect ratio measurements. Cells of the indicated strains were imaged by phase contrast microscopy and their aspect ratio (length/width) was measured as in **Figure 3.**

bioRxiv preprint doi: https://doi.org/10.64898/2026.03.13.711678; this version posted March 14, 2026. The copyright holder for this preprint (which was not certified by peer review) is the author/funder, who has granted bioRxiv a license to display the preprint in perpetuity. It is made available under aCC-BY 4.0 International license.

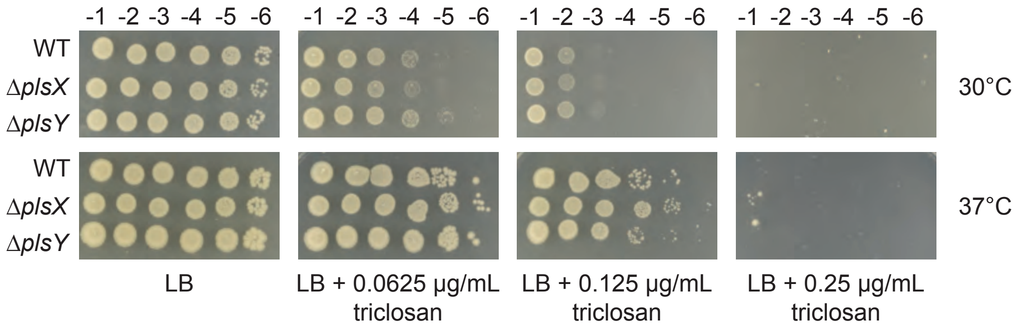

Figure S4: Loss of *plsX* does not confer a resistance to triclosan. Serial dilutions of MG1655, Δ*plsX*, and Δ*plsY* cells were spotted on LB supplemented with various concentrations of triclosan at either 30°C (**top**) or 37°C (**bottom**) for 16 hours.

bioRxiv preprint doi: https://doi.org/10.64898/2026.03.13.711678; this version posted March 14, 2026. The copyright holder for this preprint (which was not certified by peer review) is the author/funder, who has granted bioRxiv a license to display the preprint in perpetuity. It is made available under aCC-BY 4.0 International license.

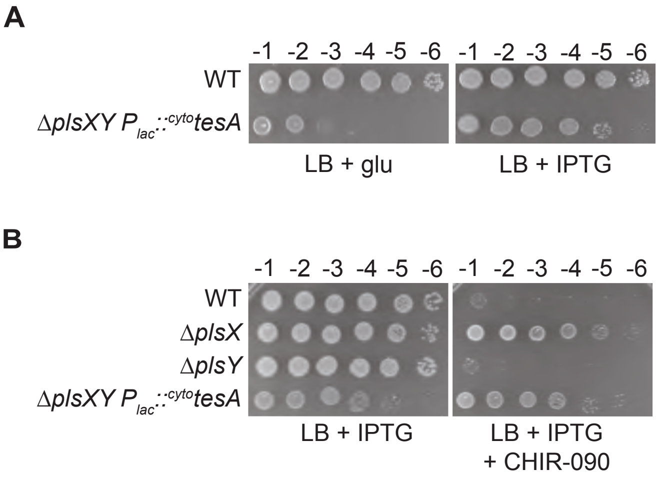

Figure S5: [cyto]TesA production rescues Δ*plsXY* synthetic lethality without influencing resistance to CHIR-090. A. Cells of the indicated strains were spotted on LB with 0.2 % glucose (left, glu) or LB with IPTG (right, 50 µM) and grown at 30°C for 17 hours before imaging. **B.** Cells of the indicated strains were spotted on LB with IPTG (1 mM) and CHIR-090 (0.075 µg/mL) as indicated. Plates were incubated at 30°C for 17 hours before imaging. In Δ*plsXY* cells [cyto]TesA was produced from pTGD35 [P_{lac}:: [cyto]*tesA*].

bioRxiv preprint doi: https://doi.org/10.64898/2026.03.13.711678; this version posted March 14, 2026. The copyright holder for this preprint (which was not certified by peer review) is the author/funder, who has granted bioRxiv a license to display the preprint in perpetuity. It is made available under aCC-BY 4.0 International license.

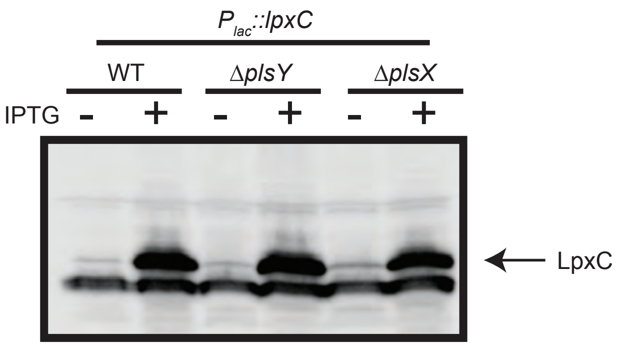

Figure S6: Quantification of LpxC overproduction. MG1655, Δ*plsX*, and Δ*plsY* containing pPR115 [P_{lac}::*lpxC*] were grown with and without IPTG (1 mM) before extracts were prepared and LpxC was detected by immunoblotting with anti-LpxC antibodies. The band corresponding to LpxC is indicated by the arrow.

bioRxiv preprint doi: https://doi.org/10.64898/2026.03.13.711678; this version posted March 14, 2026. The copyright holder for this preprint (which was not certified by peer review) is the author/funder, who has granted bioRxiv a license to display the preprint in perpetuity. It is made available under aCC-BY 4.0 International license.

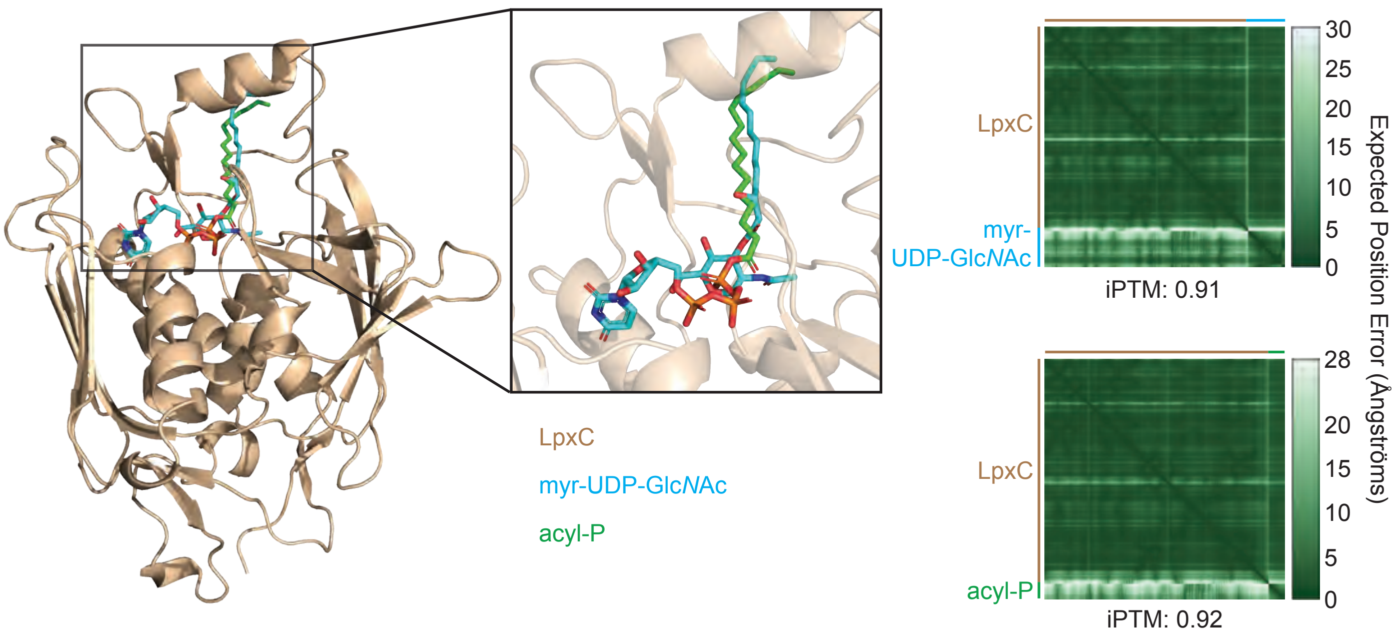

Figure S7: *E. coli* LpxC modeled with native substrate and acyl-P. Predicted structure of E. coli LpxC (beige) interacting with its native substrate UDP-3-O-(R-3-hydroxymyristoyl)-N-acetylglucosamine ("myr-UDP-GlcNAc", cyan) or palmitoyl-phosphate ("acyl-P"; green). The box shows a zoomed in view of the active site of LpxC. The prediction was modeled with AlphaFold 3. Predicted Alignment Error plots depicting the confidence of each molecule's interaction with LpxC are shown to the right.

bioRxiv preprint doi: https://doi.org/10.64898/2026.03.13.711678; this version posted March 14, 2026. The copyright holder for this preprint (which was not certified by peer review) is the author/funder, who has granted bioRxiv a license to display the preprint in perpetuity. It is made available under aCC-BY 4.0 International license.

Table S1: Strains used in this study.

**Strain Name**	**Genotype**	**Source/Reference**
DH5a(λpir)	F- hsdR17 deoR recA1 endA1 phoA supE44 thi-1 gyrA96 relA1 Δ(lacZYA-argF)U169 ø80dlacZΔM15 lpir	Lab strain
TB10	rph1 ilvG rfb-50 λΔcro-bio nad::Tn10	(2)
TB28/pTB102	rph1 ilvG rfb-50 ΔlacIZYA<>frt + pTB102	(3)
SM10	Kan^R thi-1 thr leu tonA lacY supE recA::RP4-2-Tc::Mu attl::pir	Lab strain
MG1655	rph1 lvG rfb-50	(4)
EMF229	TB10 Δ*plsX(Δ1-248)::Kan*^{R}	This study
EMF230	MG1655 Δ*plsX(Δ1-248)::Kan*^{R}	This study
EMF237	MG1655 Δ*plsX(Δ1-248):FRT*	This study
EMF229	TB10 Δ*plsX(Δ1-248)::Kan*^{R}	This study
TGD16	MG1655 Δ*plsY:FRT*	This study
PR5	MG1655 *mreC(R292H) yrdE::Kan*^{R}	(5)
EMF193/pPR11	MG1655 yejM586STOP(WT) FRT downstream + pPR11	This study
EMF193/pPR49	MG1655 yejM586STOP(WT) FRT downstream + pPR49	This study
MG1655/pPR66	MG1655 + pPR66	(6)
MG1655/pPR111	MG1655 + pPR111	(6)
MG1655/pEMF111	MG1655 + pEMF111	(6)
TGD333	MG1655 Δ*yabI::FRT*	This study
EM11	MG1655 Δ*sanA::FRT*	This study
TGD413	MG1655 Δ*lpp::FRT*	This study
TGD387	MG1655 Δ*wzzE::FRT*	This study
TGD429	MG1655 Δ*yhdP::Cam*^{R}	This study
TGD433	MG1655 Δ*damX::FRT*	This study
TGD452	MG1655 Δ*bipA::FRT*	This study
TGD453	MG1655 Δ*ybjG::FRT*	This study
TGD454	MG1655 Δ*dsbA::FRT*	This study
PR5/pPR66	PR5 + pPR66	(6)
PR5/pPR111	PR5 + pPR111	(6)
PR5/pEMF111	PR5 + pEMF111	(6)
EMF239	PR5 Δ*plsX::FRT*	This study
TGD344	PR5 Δ*yabI::FRT*	This study
TGD399	PR5 Δ*sanA::FRT*	This study
TGD400	PR5 Δ*lpp::FRT*	This study
TGD401	PR5 Δ*wzzE::FRT*	This study
TGD445	PR5 Δ*yhdP::Cam*^{R}	This study
TGD456	PR5 Δ*damX::FRT*	This study
TGD457	PR5 Δ*bipA::FRT*	This study
TGD458	PR5 Δ*ybjG::FRT*	This study
TGD459	PR5 Δ*dsbA::FRT*	This study
EMF262	PR5 Δ*plsY::FRT*	This study

bioRxiv preprint doi: https://doi.org/10.64898/2026.03.13.711678; this version posted March 14, 2026. The copyright holder for this preprint (which was not certified by peer review) is the author/funder, who has granted bioRxiv a license to display the preprint in perpetuity. It is made available under aCC-BY 4.0 International license.

MG1655/pEMF191	MG1655 + pEMF191	This study
TGD434	MG1655 + pTGD160	This study
EMF237/pEMF191	EMF237 + pEMF191	This study
TGD416	EMF237 + pTGD160	This study
MG1655/pPR115	MG1655 + pPR115	This study
EMF237/pPR111	EMF237 + pPR111	This study
EMF237/pPR115	EMF237 + pPR115	This study
TGD16/pPR111	TGD16 + pPR111	This study
TGD16/pPR115	TGD16 + pPR115	This study
SM10/pTGD89	SM10 + pTGD89	This study
EMF191	MG1655 Kan^R cassette downstream of *yejM*	This study
TGD325	Δ*plsY:Kan yejM(WT)* with FRT scar downstream + attHK022::pTGD48	This study
TGD326	Δ*plsY:Kan yejM(P143F)* with FRT scar downstream + attHK022::pTGD48	This study
TGD279	MG1655 *yejM(P143F)* with Kan^R cassette downstream	This study
TGD280	EMF237 *yejM(P143F)* with Kan^R cassette downstream	This study
TGD281	Δ*plsX(Δ1-248)::FRT* Δ*plsY:FRT* + pTGD35	This study
TGD291	PR5 *yejM(P143F)* with Kan^R cassette downstream	This study
TGD292	Δ*plsX(Δ1-248)::FRT yejM(P143F)* with FRT downstream *mreC(R292H) yrdE:Kan*	This study
TGD293	PR5 + pTGD35	This study
TGD294	Δ*plsX(Δ1-248)::FRT mreC(R292H) yrdE:Kan* + pTGD35	This study
TGD439	MG1655 + pTGD163	This study
TGD440	MG1655 + pTGD164	This study
TGD441	EMF237 + pTGD163	This study
TGD442	EMF237 + pTGD164	This study
TGD54	MG1655 attHK022::pTGD32 + pEMF35	This study
TGD55	MG1655 attHK022::pTGD32 + pEMF55	This study
TGD174	MG1655 attHK022::pTGD32 + pTGD73	This study
TGD94	MG1655 Δ*plsX(Δ1-248)::Kan^R* Δ*plsY::FRT* + pTGD1 + attHK022::pTGD48	This study
TGD129	MG1655 Δ*plsX(Δ1-248)::Kan^R* Δ*plsY::FRT* + pTGD1 + attHK022::pTGD54	This study
TGD130	MG1655 Δ*plsX(Δ1-248)::Kan* Δ*plsY::FRT* + pTGD1 + attHK022::pTGD56	This study

bioRxiv preprint doi: https://doi.org/10.64898/2026.03.13.711678; this version posted March 14, 2026. The copyright holder for this preprint (which was not certified by peer review) is the author/funder, who has granted bioRxiv a license to display the preprint in perpetuity. It is made available under aCC-BY 4.0 International license.

TableS2: Plasmids used in this study.

Plasmid	Relevant Features	Origin	Source/Reference
pCP20	Helper plasmid to cure antibiotic cassettes that were generated through lambda red recombineering	pSC101(ts)	(7)
pKD3	Recombineering template containing a chloramphenicol cassette flanked by FRT sites	R6K	(7)
pKD4	FRT-kan-FRT, used for lambda red recombineering	R6K	(7)
pKD13	Recombineering template containing a kanamycin cassette flanked by FRT sites	R6K	(7)
pTB102	Cam^R, Plasmid encodes PR::intHK022, a helper plasmid for integrating attHK022 containing CRIM vectors.	pSC101(ts)	(3)
pMT23	Tet^R, P_{lac}::*nlpD*	pBR/colE1	This study
pNG66	Tet^R, P*lac*::^{Pa}*ponB*-^{Pa}*lpoP*, attHK022 CRIM vector	R6K	(8)
pCM6	Cam^R, P_{ara}::*empty*	pACYC/p15A	(9)
pPR11	Cam^R, P_{tac}::*mreCD*	pBR/colE1	(10)
pPR49	Cam^R, P_{tac}::*mreC(R292H)D*	pBR/colE1	(5)
pPR66	Cam^R, P_{lac}::*empty*	pBR/colE1	(6)
pPR111	Cam^R, P_{lac}::*native RBS LpxC*	pBR/colE1	(6)
pEMF111	Cam^R, P_{lac}::*pbp2(L61R)-rodA*	pBR/colE1	(6)
pEMF191	Cam^R, P_{lac}::*syn op RBS* plsX*	pBR/colE1	This study
pTGD157	Cam^R, P_{lac}::*syn op RBS plsX(R80A)*	pBR/colE1	This study
pTGD160	Cam^R, P_{lac}:: *syn op RBS plsX(R80A R127A)*	pBR/colE1	This study
pPR115	Cam^R, P_{lac}::*native RBS LpxC(H265A, ΔC5)*	pBR/colE1	(11)
pDS132	Cam^R, vector template for allelic exchange, SacB	R6K	(12)
pTGD76	Cam^R, *~yejL-yejM-Kan^R~* chromosomal fragment from EMF191, SacB	R6K	This study
pTGD89	Cam^R, *~yejL-yejM(P143F)-Kan^R~* chromosomal fragment from EMF191, SacB	R6K	This study
pTGD35	Tet^R, P_{lac}::syn op RBS *tesA-cyto*	pBR/colE1	This study
pTGD163	Cam^R, P_{lac}::*syn op RBS PlsX-ALFA*	pBR/colE1	This study
pTGD164	Cam^R, P_{lac}:: *syn op RBS PlsX(R80A R127A)-ALFA*	pBR/colE1	This study
pTGD32	Tet^R, P_{tac}::*syn op RBS plsY-mScarlet*, attHK022 CRIM vector	R6K	This study
pEMF35	Tet^R, P_{ara}::*syn op RBS popZ-H3H4-msfGFP-yejM*	pBR/colE1	(6)
pEMF38	Cam^R, P_{tac}::*syn op RBS ftsH-mScarlet,* attHK022 CRIM vector	pBR/colE1	(6)
pEMF55	Cam^R, P_{ara}::*syn op RBS popZ-ftsH-H3H4-msfGFP, syn op RBS yejM*	pBR/colE1	(6)
pTGD73	Cam^R, P_{ara}::*syn op RBS popZ-H3H4-msfGFP-yejM(P143F)*	pBR/colE1	This study

bioRxiv preprint doi: https://doi.org/10.64898/2026.03.13.711678; this version posted March 14, 2026. The copyright holder for this preprint (which was not certified by peer review) is the author/funder, who has granted bioRxiv a license to display the preprint in perpetuity. It is made available under aCC-BY 4.0 International license.

pTGD1	Cam^R, P_{ara}::*native RBS plsY*	pACYC	This study
pTGD48	Tet^R, P_{lac}::*syn op RBS plsY-ALFA*, attHK022 CRIM vector	R6K	This study
pTGD54	Tet^R, P_{lac}::*syn op RBS plsY(G107P)-ALFA*, attHK022 CRIM vector	R6K	This study
pTGD56	Tet^R, P_{lac}::*syn op RBS plsY(N180H)-ALFA*, attHK022 CRIM vector	R6K	This study

*All instances of “syn op RBS” refer to the synthetically optimized ribosomal binding sequence: tttaagaaggagatatacat

bioRxiv preprint doi: https://doi.org/10.64898/2026.03.13.711678; this version posted March 14, 2026. The copyright holder for this preprint (which was not certified by peer review) is the author/funder, who has granted bioRxiv a license to display the preprint in perpetuity. It is made available under aCC-BY 4.0 International license.

Table S3: Primers used in this study.

**PlsX_Kan_F** —<br>AGGCAACTGGGGAAAGACCAAACCGGGCGGCGACGATACCTGTAGGCTGGAGCTGCTTCG<br>F primer used to generate strain EMF229
**PlsX_kan_R** —<br>GTTTTTTCCCTTCACCCTGAGATTTCAGCAGAGAAAGGAAATTCCGGGGATCCGTCGACC<br>R primer used to generate strain EMF229:
**strongRBS_plsX_F** —<br>CCCCTCTAGATTTAAGAAGGAGATATACATTTGACACGTCTAACCCTGGC<br>F primer used to generate pEMF191
**plsX_R** —<br>TGATAAGCTTACTACCGCAGAGTTCCGCTTTTG<br>R primer used to generate pEMF191
**TGD197** —<br>GCATAGGGTTTGCAGAATCCCTGCTTCGTCCATTTGACAGGCACATTATGATTCCGGGGATCCGTCGACC<br>F primer for replacing MG1655 chromosomal *yabI* with a Kan^R cassette
**TGD198** —<br>GCATCAGGCAACCCGCACAAGACTATCTCCTAAACCCCAACCACTTTACGTGTAGGCTGGAGCTGCTTCG<br>R primer for replacing MG1655 chromosomal *yabI* with a Kan^R cassette
**TGD229** —<br>AGAAATCGTGGTGGCAGCCCCAATTTAACCAAATAAATGAGGATGTGATGATTCCGGGGATCCGTCGACC<br>F primer for replacing MG1655 chromosomal *wzzE* with Kan^R cassette
**TGD230** —<br>GCGCCTTTGCGCTCACCGCAGCAGTGTTGCTATTTCGAGCAACGGCGGGTTGTAGGCTGGAGCTGCTTCG<br>R primer for replacing MG1655 chromosomal *wzzE* with Kan^R cassette
**TGD271** —<br>cggcaaagggtttttgagtcacatttttagcagacaaggagtgacgggtggccatggtccatatgaatatcctcc<br>F primer to replace MG1655 chromosomal *yhdP* with Cam^R cassette
**TGD 272** —<br>gattggggcaattacgcgccctcgtcaaatcattgcgctttttctttacggcgattgtgtaggctggagc<br>R primer to replace MG1655 chromosomal *yhdP* with Cam^R cassette
**TGD279** —<br>taatccgtgtacaataacgcgctatttctaatgcctgaggcaaagttgtgattccggggatccgtcgacc<br>F primer to replace MG1655 chromosomal *bipA* with Kan^R cassette
**TGD280** —<br>ggcaggtttttatgactaaaaaacgaaattaatcgtctttcggtgcgcgTGTAGGCTGGAGCTGCTTCG<br>R primer to replace MG1655 chromosomal *bipA* with Kan^R cassette
**TGD277** —<br>tattctcaacgacttgcctgtattggctcccttttaatcactttgcgtcgggaagttatgATTCCGGGGATCCGTCGACC<br>F primer to replace MG1655 chromosomal *ybjG* with Kan^R cassette
**TGD278** —<br>ttgaagcgagccgctcaatactacactttttagcagagatcagtcacgcacccagcctttTGTAGGCTGGAGCTGCTTCG<br>R primer to replace MG1655 chromosomal *ybjG* with Kan^R cassette

bioRxiv preprint doi: https://doi.org/10.64898/2026.03.13.711678; this version posted March 14, 2026. The copyright holder for this preprint (which was not certified by peer review) is the author/funder, who has granted bioRxiv a license to display the preprint in perpetuity. It is made available under aCC-BY 4.0 International license.

**yejM586STOP_F —**<br>GCAAGTGCTGACAGACGAGAAGCGTTTTATCGCTAACTGATTGTGTAGGCTGGAGCTGCT<br>F primer used to amplify the Kan cassette from pKD4 to generate strain EMF127
**yejM_kan_truncation_R —**<br>GATTGCAAGTAAGATATTTCGCTAACTGATTTATAATTAACATATGAATATCCTCCTTAG<br>R primer used to amplify the Kan^R cassette from pKD4 to generate strain EMF127
**TGD169 —**<br>GCAAGCTATCgcgGCCAGTCGTGGG<br>F primer to mutagenize pEMF191(*plsX*) to generate pTGD157(*plsX(R80A)*)
**TGD170 —**<br>GAAGGCCGGGCATCA<br>R primer to mutagenize pEMF191(*plsX*) to generate pTGD157(*plsX(R80A)*)
**TGD171 —**<br>GGGGATTGAGgcgCCGGCGCTGG<br>F primer to mutagenize pTGD157(*plsX(R80A)*) to generate pTGD160(*plsX(R80A R127A)*)
**TGD172 —**<br>TCCAGGGGCTTGAGTAATAATT<br>R primer to mutagenize pTGD157(*plsX(R80A)*) to generate pTGD160(*plsX(R80A R127A)*)
**TGD96 —**<br>GTTAAACGCGAAAGCGCAAAACCGgagctctcccgggaattccac<br>Gibson assembly R backbone primer for cloning *yejM* with downstream Kan^R from EMF191 into pDS132 with homology to TGD102 to generate pTGD76
**TGD97 —**<br>tgcctgcttgccgaatatcatggtgtctagaggatcgatcctttttaacccatcac<br>Gibson assembly F backbone primer for cloning *yejM* with downstream Kan^R from EMF191 into pDS132 with homology to TGD102 to generate pTGD76
**TGD102 —**<br>gtggaattcccgggagagctcCGGTTTTGCGCTTTCGCGTTTAAC<br>F primer for *yejM* with downstream Kan^R allelic exchange in EMF191 with homology to pDS132 backbone with homology to TGD96 to generate pTGD76
**TGD103 —**<br>gtgatgggttaaaaaggatcgatcctctagacaccatgatattcggcaagcaggca<br>R primer for *yejM* with downstream Kan^R allelic exchange in EMF191 with homology to pDS132 backbone with homology to TGD97 to generate pTGD76
**TGD88 —**<br>CATCAGCGTGtttGTTATTTTATTGCTTG<br>F primer to mutagenize pTGD76(*~yejL-yejM-Kan^R~* chromosomal fragment from EMF191) to generate pTGD89(*~yejL-yejM(P143F)-Kan^R~)*
**TGD89 —**<br>AACATCAGCTGCCAG<br>R primer to mutagenize pTGD76(*~yejL-yejM-Kan^R~* chromosomal fragment from EMF191) to generate pTGD89(*~yejL-yejM(P143F)-Kan^R~)*
**TGD50 —**<br>GCTATCTAGATTAAGAAGGAGATATACATatgGCGGACACGTTATTGATTCTGGG

bioRxiv preprint doi: https://doi.org/10.64898/2026.03.13.711678; this version posted March 14, 2026. The copyright holder for this preprint (which was not certified by peer review) is the author/funder, who has granted bioRxiv a license to display the preprint in perpetuity. It is made available under aCC-BY 4.0 International license.

F primer to amplify MG1655 chromosomal *tesA* with a XbaI sequence, a synthetically optimized RBS, and without its signal sequence to generate *tesA-cyto*[62]
**TGD49** — GCTAAAGCTTttaTGAGTCATGATTTACTAAAGGCTGCAACTG R primer to amplify MG1655 chromosomal *tesA* with a HindIII sequence
**TGD287** — cgccgccgcctgaccgaaccgtgaTAAGCTTATCACCGATACGCG F primer to add Alfa tag to pEMF191 and pTGD160 to make pTGD163 and pTGD164, respectively
**TGD288** — cagttcttcttccaggcggctcggCCGCAGAGTTCCGCTTTTG F primer to add Alfa tag to pEMF191 and pTGD160 to make pTGD163 and pTGD164, respectively
**TGD38** — CCCCTCTAGAAATAATTTTGTTTAACTTTAAGAAGGAGATATACATATGAGTGCAATCGCGCCTGG F Gibson primer to amplify chromosomal *plsY* with homology to pEMF38 to create pTGD32
**TGD39** — CCCTCGAGACCAGAGGATCCCTCGGGATCCTTTTCGCGC R Gibson primer to amplify chromosomal *plsY* with homology to pEMF38 to create pTGD32
**TGD40** — GGATCCTCTGGTCTCGAGGG F Gibson primer to amplify pEMF38 backbone with homology to *plsY* to create pTGD32
**TGD41** — CATATGTATATCTCCTTCTTAAAGTTAAACAAAATTATTTCTAGAGGGG R Gibson primer to amplify pEMF38 backbone with homology to *plsY* to create pTGD32
**TGD88** — CATCAGCGTGtttGTTATTTTATTGCTTG F primer to mutagenize *yejM* in pEMF35 to *yejM(P143F)* to create pTGD73
**TGD89** — AACATCAGCTGCCAG R primer to mutagenize *yejM* in pEMF35 to *yejM(P143F)* to create pTGD73
**TGD7** — GCTATCTAGAAATTTTCAAAACGGAACAGCTTatgAGTGCAATCG F primer to amplify MG1655 chromosomal *plsY* with native RBS and a XbaI sequence
**TGD8** — GCTAAAGCTTtcaCTCGGGATCCTTTTCGCG R primer to amplify MG1655 chromosomal *plsY* with a HindIII sequence
**TGD66** — GCTATCTAGATTTAAGAAGGAGATATACATATGAGTGCAATCGCGCCTGG F primer to amplify MG1655 chromosomal *plsY* with a synthetically optimized RBS and XbaI sequence
**TGD68** — GCTAAAGCTTTCAcggttcggtcaggcggcggcgcagttcttcttccaggcggctcggCTCGGGATCCTTTTCGCGCTT R primer to amplify MG1655 chromosomal *plsY* with a C-terminal Alfa tag and HindIII sequence
**TGD75** — AGGAGGAAAAccgGTTGCTACCGCTTTTG F primer to mutagenize *plsY* in pTGD48 to generate *plsY(G107P)* in pTGD54

bioRxiv preprint doi: https://doi.org/10.64898/2026.03.13.711678; this version posted March 14, 2026. The copyright holder for this preprint (which was not certified by peer review) is the author/funder, who has granted bioRxiv a license to display the preprint in perpetuity. It is made available under aCC-BY 4.0 International license.

**TGD76** — TTAAATCCGAAGAAAACGG<br>R primer to mutagenize *plsY* in pTGD48 to generate *plsY(G107P)* in pTGD54
**TGD79** — TCATCATGACcatATCCAACGTCTG<br>F primer to mutagenize *plsY* in pTGD48 to generate *plsY(N180H)* in pTGD56
**TGD80** — CGCAGCAGGATCAGG<br>R primer to mutagenize *plsY* in pTGD48 to generate *plsY(N180H)* in pTGD56
**CRIM-P2** — ACTTAACGGCTGACATGG<br>Primer to quantify how many times a CRIM vector was integrated into the chromosome
**CRIM-P3** — ACGAGTATCGAGATGGCA<br>Primer to quantify how many times a CRIM vector was integrated into the chromosome
**attHK-P1** — GGAATCAATGCCTGAGTG<br>Primer to quantify how many times a CRIM vector was integrated into the chromosome at attHK022
**attHK-P4** — GGCATCAACAGCACATTC<br>Primer to quantify how many times a CRIM vector was integrated into the chromosome at attHK022
**ZL110** — ATTATCCACAGATTCATCGTTGAACACGAATTTTCAAAACGGAACAGCTTatgATTCCGGGGATCCGTCGACC<br>F primer for replacing MG1655 chromosomal *plsY* with a Keio-like Kan cassette
**ZL111** — GCTATCAGGTCATGTAGATCCACCAGAAAtcaCTCGGGATCCTTTTCGCGTGTAGGCTGGAGCTGCTTCG<br>R primer for replacing MG1655 chromosomal *plsY* with a Keio-like Kan cassette

bioRxiv preprint doi: https://doi.org/10.64898/2026.03.13.711678; this version posted March 14, 2026. The copyright holder for this preprint (which was not certified by peer review) is the author/funder, who has granted bioRxiv a license to display the preprint in perpetuity. It is made available under aCC-BY 4.0 International license.

Table S4: Strain construction information.

Strain	Relevant Construction Information
EMF127	The Kan^R cassette from pKD4 was amplified with primers yejM586STOP_F (GCAAGTGCTGACAGACGAGAAGCGTTTTATCGCTAACTGATTGTGTAGGCTGGAGCTGCT) and yejM_kan_truncation_R (GATTGCAAGTAAGATATTTCGCTAACTGATTTATAATTAACATATGAATATCCTCCTTAG). The PCR product was purified, electroporated into TB10/pEMF31, and the Kan^R cassette was integrated downstream of yejM by lambda red recombineering. The strain was confirmed by PCR.
EMF229	The Kan^R cassette was amplified from pKD13 using primers PlsX_Kan_F (AGGCAACTGGGGAAAGACCAAACCGGGCGGCGACGATACCTGTAGGCTGGAGCTGCTTCG)And PlsX_Kan_R (GTTTTTTCCCTTCACCCTGAGATTTCAGCAGAGAAAGGAAATTCCGGGGATCCGTCGACC). The PCR product was purified, electroporated into TB10, and the Kan^R cassette was intergrated at the plsX locus by lambda red recombineering. The strain was confirmed by PCR.
EMF230	The plsX(Δ1-248)::Kan^R allele from EMF229 was transduced into MG1655 via P1 transduction and confirmed via PCR.
EMF237	The Kan^R cassette from EMF230 was cured via Pcp20 as described previously and confirmed via PCR
TGD16	MG1655 chromosomal *plsY* was replaced by a Kan^R cassette via recombineering with ZL110 and ZL111 and then cured with pCP20, yielding Δ*plsY::FRT*. The strain was confirmed by PCR.
EMF193/pPR11	EMF193 was transformed with pPR11
EMF193/pPR49	EMF193 was transformed with pPR49
MG1655/pPR66	MG1655 was transformed with pPR66
MG1655/pPR111	MG1655 was transformed with pPR11
MG1655/pEMF111	MG1655 was transformed with pEMF111
TGD333	MG1655 chromosomal *yabI* was replaced by a Kan^R cassette via recombineering with TGD197 and TGD198 and then cured with pCP20, yielding Δ*yabI::FRT*. The strain was confirmed by PCR.
EM11	MG1655 was transduced with a Δ*sanA:Kan^R* P1 lysate and then cured with pCP20 to yield Δ*sanA::FRT*. The strain was confirmed by PCR.
TGD413	MG1655 was transduced with a Δ*lpp:Kan^R* P1 lysate and then cured with pCP20 to yield Δ*lpp::FRT*. The strain was confirmed by PCR.
TGD387	MG1655 chromosomal *wzzE* was replaced by a Kan^R cassette via recombineering with TGD229 and TGD230 and then cured with pCP20, yielding Δ*wzzE::FRT*. The strain was confirmed by PCR.
TGD429	MG1655 chromosomal *yhdP* was replaced by a Cam^R cassette via recombineering with TGD271 and TGD272 yielding Δ*yhdP::Cam^R* The strain was confirmed by PCR.
TGD433	MG1655 was transduced with a Δ*damX:Kan^R* P1 lysate and then cured with pCP20 to yield Δ*damX::FRT*. The strain was confirmed by PCR.
TGD452	MG1655 chromosomal *bipA* was replaced by a Kan^R cassette via recombineering with TGD279 and TGD280 and then cured with pCP20, yielding Δ*bipA::FRT*. The strain was confirmed by PCR.
TGD453	MG1655 chromosomal *ybiG* was replaced by a Kan^R cassette via recombineering with TGD277 and TGD278 and then cured with pCP20, yielding Δ*ybjG::FRT*. The strain was confirmed by PCR.
TGD454	MG1655 was transduced with a Δ*dsbA::Kan^R* P1 lysate and then cured with pCP20 to yield Δ*dsbA::FRT*. The strain was confirmed by PCR.
PR5/pPR66	MG1655/pPR66 was transduced with a *mreC(R292H) yrdE::Kan^R* P1 lysate

bioRxiv preprint doi: https://doi.org/10.64898/2026.03.13.711678; this version posted March 14, 2026. The copyright holder for this preprint (which was not certified by peer review) is the author/funder, who has granted bioRxiv a license to display the preprint in perpetuity. It is made available under aCC-BY 4.0 International license.

PR5/pPR111	MG1655/pPR111 was transduced with a *mreC(R292H) yrdE::Kan^R* P1 lysate
PR5/pEMF111	MG1655/pEMF111 was transduced with a *mreC(R292H) yrdE::Kan^R* P1 lysate
EMF239	EMF237 was transduced with *mreC(R292H) yrdE::Kan^R* P1 lysate
TGD344	TGD333 was transduced with *mreC(R292H) yrdE::Kan^R* P1 lysate
TGD399	EM11 was transduced with *mreC(R292H) yrdE::Kan^R* P1 lysate
TGD400	TGD413 was transduced with a *mreC(R292H) yrdE::Kan^R* P1 lysate
TGD401	TGD387 was transduced with a *mreC(R292H yrdE::Kan^R* P1 lysate
TGD445	TGD429 was transduced with a *mreC(R292H) yrdE::Kan^R* P1 lysate
TGD456	TGD433 was transduced with a *mreC(R292H) yrdE::Kan^R* P1 lysate
TGD457	TGD452 was transduced with a *mreC(R292H) yrdE::Kan^R* P1 lysate
TGD458	TGD453 was transduced with a *mreC(R292H) yrdE::Kan^R* P1 lysate
TGD459	TGD454 was transduced with a *mreC(R292H) yrdE::Kan^R* P1 lysate
EMF262	TGD16 was transduced with a *mreC(R292H) yrdE::Kan^R* P1 lysate
MG1655/pEMF191	MG1655 was transformed with pEMF191
TGD434	MG1655 was transformed with pTGD160
EMF237/pEMF191	EMF237 was transformed with pEMF191
TGD416	EMF237 was transformed with pTGD160
MG1655/pPR115	MG1655 was transformed with pPR115
EMF237/pPR111	EMF237 was transformed with pPR111
EMF237/pPR115	EMF237 was transformed with pPR115
TGD16/pPR111	TGD was transformed with pPR111
TGD16/pPR115	TGD was transformed with pPR115
SM10/pTGD89	SM10 was transformed with pTGD89
EMF191	The Kan^R cassette downstream of yejM was transduced from strain EMF127 to strain MG1655 by P1 transduction. Transductants were elected for on LB + kanamycin and confirmed by PCR.
TGD325	EMF191 was transformed with pCP20 and had its Kan^R cassette removed before being transduced with a *ΔplsY::Kan^R* P1 lysate and then an attHK022::pTGD48 P1 lysate yielding *ΔplsY::Kan attHK022::pTGD48 yejM(WT)* with a downstream FRT scar
TGD326	TGD279 was transformed with pCP20 and had its Kan^R cassette removed before being transduced with a *ΔplsY::Kan^R* P1 lysate and then an attHK022::pTGD48 P1 lysate yielding *ΔplsY::Kan attHK022::pTGD48 yejM(P143F)* with a downstream FRT scar
TGD279	MG1655 was transduced with a *yejM(P143F)* Kan^R downstream P1 lysate
TGD280	EMF237 was transduced with a *yejM(P143F)* Kan^R downstream P1 lysate
TGD281	EMF237 was transformed with pTGD35, transduced with a *ΔplsY::Kan^R* P1 lysate, and then transformed with pCP20 to cure the Kan cassette yielding *ΔplsX:FRT ΔplsY::FRT* with pTGD35
TGD291	TGD279 was transduced with a *mreC(R292H) yrdE::Kan^R* P1 lysate
TGD292	TGD280 was transduced with a *mreC(R292H) yrdE::Kan^R* P1 lysate
TGD293	MG1655 possessing pTGD35 was transduced with a *mreC(R292H) yrdE::Kan^R* P1 lysate
TGD294	EMF237 possessing pTGD35 was transduced with a *mreC(R292H) yrdE::Kan^R* P1 lysate
TGD439	MG1655 was transformed with pTGD163
TGD440	MG1655 was transformed with pTGD164
TGD441	EMF237 was transformed with pTGD163
TGD442	EMF237 was transformed with pTGD164

bioRxiv preprint doi: https://doi.org/10.64898/2026.03.13.711678; this version posted March 14, 2026. The copyright holder for this preprint (which was not certified by peer review) is the author/funder, who has granted bioRxiv a license to display the preprint in perpetuity. It is made available under aCC-BY 4.0 International license.

TGD54	MG1655 was transduced with an attHK022::pTGD32 P1 lysate and then transformed with pEMF35
TGD55	MG1655 was transduced with an attHK022::pTGD32 P1 lysate and then transformed with pEMF55
TGD174	MG1655 was transduced with an attHK022::pTGD32 P1 lysate and then transformed with pEMF73
TGD94	TGD16 was transformed with pTGD1 then transduced with a ∆plsX(∆1-248)::Kan^R P1 lysate and an attHK022::pTGD48 P1 lysate to yield *∆plsY::FRT ∆plsX(∆1-248):Kan^R* attHK022::pTGD48 + pTGD1
TGD129	TGD16 was transformed with pTGD1 then transduced with a ∆plsX(∆1-248)::Kan^R Kan P1 lysate and an attHK022::pTGD54 P1 lysate to yield *∆plsY::FRT ∆plsX(∆1-248):Kan^R* attHK022::pTGD54 + pTGD1
TGD130	TGD16 was transformed with pTGD1 then transduced with a ∆plsX(∆1-248)::Kan^R P1 lysate and an attHK022::pTGD56 P1 lysate to yield *∆plsY::FRT ∆plsX(∆1-248):Kan^R* attHK022::pTGD56 + pTGD1

bioRxiv preprint doi: https://doi.org/10.64898/2026.03.13.711678; this version posted March 14, 2026. The copyright holder for this preprint (which was not certified by peer review) is the author/funder, who has granted bioRxiv a license to display the preprint in perpetuity. It is made available under aCC-BY 4.0 International license.

Table S5: Plasmid construction information.

Plasmid	Relevant Construction Information
pEMF191	Primers strongRBS_plsX_F and plsX_R were used to amplify plsX from genomic DNA. The PCR product was purified and inserted into pPR66 using the restriction enzymes xbaI and hindIII.
pTGD157	pEMF191(*plsX*) was mutagenized with primers TGD169 and TGD170 to yield pTGD157(*plsX(R80A)*)
pTGD160	pTGD157(*plsX(R80A)*) was mutagenized with primers TGD171 and TGD172 to yield pTGD160(*plsX(R80A R127A)*)
pTGD76	Primers TGD96 and TGD97 were used to amplify a MG1655 chromosomal fragment of *yejM* and the surrounding genes, including a downstream Kan^R cassette, from EMF191 with homology to the pDS132 backbone. Primers TGD102 and TGD103 were then used to amplify the pDS132 backbone with homology to the chromosomal fragment amplified by TGD96 and TGD97. Together, the resulting PCR products were used in a Gibson reaction to yield pTGD76
pTGD89	Primers TGD88 and TGD89 were used to mutagenize the *yejM* in pTGD76 to *yejM(P143F)*, yielding pTGD89
pTGD35	Primers TGD49 and TGD50 were used to amplify MG1655 chromosomal *tesA* and remove the native signal sequence, add a synthetically optimized RBS, and add XbaI/HindIII cut sites. This PCR product was XbaI/HindIII digested and then ligated into a XbaI/HindIII digested pMT23 backbone to yield pTGD35
pTGD163	An ALFA tag was added to pEMF191(*plsX)* using primers TGD287 and TGD288 to yield pTGD163(*plsX-ALFA)*
pTGD164	An ALFA tag was added to pTGD160(*plsX(R80A R127A))* using primers TGD287 and TGD288 to yield pTGD164(*plsX(R80A R127A)-* ALFA*)*
pTGD32	Primers TGD38 and TGD39 were used to amplify MG1655 chromosomal *plsY* with homology to the pEMF38 vector backbone. Primers TGD40 and TGD41 were used to amplify pEMF38 with homology to chromosomal *plsY*. Together, these PCR products were used in a Gibson reaction to yield pTGD32
pTGD73	Primers TGD88 and TGD89 were used to mutagenize *yejM* in pEMF35 to *yejM(P143F)* to yield pTGD73
pTGD1	Primers TGD7 and TGD8 were used to amplify MG1655 chromosomal *plsY* with the native RBS and added XbaI/HindIII cut sites. This PCR product was digested with XbaI/HindIII and ligated into a XbaI/HindIII digested pCM6 backbone to yield pTGD1
pTGD48	Primers TGD66 and TGD68 were used to amplify MG1655 chromosomal *plsY* and add a synthetically optimized RBS, a C-terminal ALFA tag, and XbaI/HindIII cut sites. This PCR product was digested with XbaI/HindIII and then ligated into a XbaI/HindIII digested pNG66 backbone to yield pTGD48
pTGD54	Primers TGD75 and TGD76 were used to mutagenize *plsY* in pTGD48 to generate *plsY(G107P)* in pTGD54
pTGD56	Primers TGD79 and TGD80 were used to mutagenize *plsY* in pTGD48 to generate *plsY(N180H)* in pTGD56

bioRxiv preprint doi: https://doi.org/10.64898/2026.03.13.711678; this version posted March 14, 2026. The copyright holder for this preprint (which was not certified by peer review) is the author/funder, who has granted bioRxiv a license to display the preprint in perpetuity. It is made available under aCC-BY 4.0 International license.

REFERENCES

1. Mendler K, Chen H, Parks DH, Lobb B, Hug LA, Doxey AC. 2019. AnnoTree: visualization and exploration of a functionally annotated microbial tree of life. Nuc Acid Res 47:4442–4448.

2. Johnson JE, Lackner LL, Hale CA, Boer PAJ de. 2004. ZipA is required for targeting of DMinC/DicB, but not DMinC/MinD, complexes to septal ring assemblies in Escherichia coli. J Bacteriol 186:2418–2429.

3. Bernhardt TG, Boer PAJ de. 2005. SlmA, a nucleoid-associated, FtsZ binding protein required for blocking septal ring assembly over Chromosomes in E. coli. Mol Cell 18:555–564.

4. Guyer MS, Reed RR, Steitz JA, Low KB. 1981. Identification of a sex-factor-affinity site in E. coli as gamma delta. Cold Spring Harbor symposia on quantitative biology 45 Pt 1:135–140.

5. Rohs PDA, Buss J, Sim SI, Squyres GR, Srisuknimit V, Smith M, Cho H, Sjodt M, Kruse AC, Garner EC, Walker S, Kahne DE, Bernhardt TG. 2018. A central role for PBP2 in the activation of peptidoglycan polymerization by the bacterial cell elongation machinery. PLOS Genet 14:e1007726.

6. Fivenson EM, Bernhardt TG. 2020. An Essential Membrane Protein Modulates the Proteolysis of LpxC to Control Lipopolysaccharide Synthesis in Escherichia coli. mBio 11:e00939-20.

7. Datsenko KA, Wanner BL. 2000. One-step inactivation of chromosomal genes in Escherichia coli K-12 using PCR products. Proc Natl Acad Sci USA 97:6640–6645.

8. Greene NG, Fumeaux C, Bernhardt TG. 2018. Conserved mechanism of cell-wall synthase regulation revealed by the identification of a new PBP activator in Pseudomonas aeruginosa. - PubMed - NCBI. Proc Natl Acad Sci USA 115:3150–3155.

9. Yunck R, Cho H, Bernhardt TG. 2015. Identification of MltG as a potential terminase for peptidoglycan polymerization in bacteria. Mol Microbiol 99:700–718.

10. Rohs PDA, Qiu JM, Torres G, Smith MD, Fivenson EM, Bernhardt TG. 2021. Identification of Potential Regulatory Domains within the MreC and MreD Components of the Cell Elongation Machinery. J Bacteriol 203:e00493-20.

11. Fivenson EM, Rohs PDA, Vettiger A, Sardis MF, Torres G, Forchoh A, Bernhardt TG. 2023. A role for the Gram-negative outer membrane in bacterial shape determination. Proc Natl Acad Sci USA120:e2301987120.

12. Philippe N, Alcaraz J-P, Coursange E, Geiselmann J, Schneider D. 2004. Improvement of pCVD442, a suicide plasmid for gene allele exchange in bacteria. Plasmid 51:246–255.
